# Differences in the learning curve of robotic transabdominal preperitoneal inguinal hernia repair according to surgeon’s robotic experience

**DOI:** 10.1007/s10029-023-02846-4

**Published:** 2023-08-17

**Authors:** L. Solaini, D. Cavaliere, G. Rocco, A. Avanzolini, D. Di Pietrantonio, G. Ercolani

**Affiliations:** 1 Department of Medical and Surgical Sciences-DIMEC, Alma Mater Studiorum-University of Bologna, Bologna, Italy; 2 General and Oncologic Surgery, Department of Surgery, Morgagni-Pierantoni Hospital, via C. Forlanini 29, 47121 Forlì, Italy; 3 General Surgery, Department of Surgery, Infermi Hospital, Faenza, Italy

**Keywords:** Hernia repair, Learning, Robot, Minimally invasive surgery, Groin

## Abstract

**Purpose:**

In this study, we aim to analyze the learning curve of each step of robotic transabdominal pre-peritoneal inguinal hernia repair (rTAPP) in two surgeons with varying degrees of expertise with the robotic platform but no experience with laparoscopic hernia repair.

**Methods:**

Data on 124 rTAPP cases performed by two surgeons were retrospectively reviewed. Cumulative sum (CUSUM) analysis was applied to visualize the learning curve of rTAPP on operation time of each step of the procedure [the peritoneal flap creation (T1), the completion of the critical view of the myopectineal orifice (T2), the mesh application (T3) and the peritoneal flap closure (T4)]. Each intraoperative and postoperative outcome was compared according to surgeon’s experience with the robotic platform and learning phase. The robotic surgeon mentored the surgeon-in-training and was present during all surgeries in his learning period.

**Results:**

The surgeon in training with the robotic platform showed a learning phase till the 20th procedure followed by a gradual improvement in performances. The expert surgeon showed a learning phase till the 35th procedure after which a constant decrease of operative time was recorded till the last procedure included. The operative times of each step of the procedures of both surgeons were significantly improved after the learning phase. In the late phase, the surgeon in training could achieve operative times in T2 and T3, which are similar to those of an experienced robotic surgeon with no experience with TAPP before the completion of the learning phase.

**Conclusions:**

In conclusion, the learning phase of rTAPP surgery may vary between 20 and 35 cases, depending on the surgeon’s experience in robotic surgery.

## Background

The use of the robotic platform has become common in inguinal hernia repair in recent years [1–3]. This may be due to the intrinsic well-established advantages offered by the robotic platform along with an improved ergonomic, which is not so obvious in the laparoscopic counterpart.

The technological characteristics of the robotic platform and its several instruments for training (e.g. double console, tele-mentoring……) may concur in reducing the learning curve (LC) of several minimally invasive procedures and, thus, reducing the LC effect on their outcomes [2, 4, 5]. Several studies about this topic have been published in the different fields of general surgery in the last decade, but, data on the LC of robotic inguinal hernia repair are scarce.

Currently, only three studies performed a structured analysis on the LC of robotic transabdominal pre-peritoneal inguinal hernia repair (rTAPP) [6–8] and according to the sample size of each series, they found different time points to define the end of the learning phases. However, in all cases, rTAPP was performed by expert surgeons in laparoscopic TAPP focusing the analyses only on the impact of the robotic platform on the procedure. This may lead to misinterpretation of LC data from rTAPP when they are compared with the laparoscopic approach for which, in all cases, the LC is referred both to the approach and the procedure.

In this study, we aim to analyze the LG of each step of rTAPP in two surgeons with varying degrees of expertise with the robotic platform but no experience with laparoscopic inguinal hernia repair.

## Methods

In this retrospective cohort study, all consecutive patients who underwent robotic inguinal hernia repair at Morgagni-Pierantoni Hospital, University of Bologna, Forlì between 1 January 2020 and 31 December 2022 were included.

This study exploited an institutional review board-approved database.

### Variables and definitions

Variables collected included sex, age and body mass index (BMI), American Society of Anaesthesiologists (ASA) score, side, type of hernia, operative time, morbidity and length of hospital stay.

Postoperative complications were recorded up to 90 days after surgery.

Primary outcome was operative time. Secondary outcomes included 90 days of postoperative morbidity and length of hospital stay.

Inguinal hernia classification by the European Hernia Society was used as a surrogate difficulty score [9].

### Participants and surgical technique

All procedures were performed by two surgeons with no experience with minimally invasive TAPP. One surgeon was expert in robotic surgery (around 80 procedures per year, mainly colorectal), while the other surgeon, who completed his residency program in general surgery in 2016 and had expertise in laparoscopic gastric and colorectal procedures, began his training with the robotic platform by performing rTAPP. The surgeon undergoing training with the robotic platform attended standard robotic training programs, while both surgeons received specialized training in performing rTAPP. This training included dedicated courses and case observation.

In summary, the inexperienced surgeon in robotic surgery began his training utilizing the simulator, participating in dry-lab and wet-lab sessions, and attending hands-on courses. Since the beginning of the robotic surgery training, the trainee surgeon has assisted in over 150 robotic procedures, primarily focusing on colorectal surgery. Approximately, segments of 20 robotic procedures were carried out, under constant supervision, before the first rTAPP in this study. The trainee surgeon functioned as the first assistant surgeon to the experienced surgeon for all rTAPP procedures incorporated in the study. Additionally, various other robotic operations were performed during the same period. The experienced robotic surgeon mentored the trainee surgeon during the learning period.

TAPP was performed according to what has already been reported in literature for the laparoscopic approach [10]. Da Vinci® Si was used till September 2022, while for the remaining procedures, Xi system was used. The Si system was docked between the patient’s legs, while the Xi system from the right side. The patient was placed in the supine position with 15-/20-degree Trendelenburg. Three 8-mm robotic trocars were inserted in the supra-umbilical region, right and left flanks on the same line.

In summary, the procedure consisted of four main steps as described elsewhere [10]: the peritoneal flap creation, the completion of the critical view of the myopectineal orifice, the mesh application and the peritoneal flap closure.

All procedures were performed using ProGrip™ Laparoscopic Self-Fixating Mesh, 15 × 10 cm (Covidien, North Haven, CT, USA).

### Statistical analysis

Outcomes and learning curves of each surgeon were compared. Continuous variables, which were presented as medians with interquartile range (IQR), were compared with the Mann–Whitney *U* test. Categorical variables were presented as frequencies with percentages, and compared by the Fisher’s exact test.

Operative times were monitored using a standard cumulative sum (CUSUM) chart [11, 12] for each surgeon. The first peak on the trend line represented conventionally the end of the learning phase and the beginning of the proficiency phase. A retrospective review of the recorded video of each procedure was performed in order to calculate the exact duration of the four main steps of TAPP. In particular, T1 was defined as the time between the first incision of the peritoneum and the visualization of Cooper ligament, T2 between visualization of Cooper ligament and the completion of the critical view of the myopectineal orifice with the full parietalization of the elements, T3 between the parietalization of the elements and the unrolling of the self-fixating mesh, and T4 between the unrolling of the mesh and the fully sutured peritoneal flap.

Bilateral hernia repairs were considered as two distinct procedures for the analyses, and the operative time was obtained by the sum of the time of the four steps per side plus the time in common to place the trocars, to dock the surgical robot’s manipulator arm unit and to close the incisions.

A *p* value < 0.05 was considered statistically significant. Data were analyzed using the StataCorp 2017 (Stata Statistical Software: Release 15, StataCorp LLC).

## Results

In total, 124 procedures were included in the analysis. Detailed characteristics of the included patients and procedures divided according to the operating surgeon were shown in Table 1.

**Table 1:** Overall patients’ characteristics and perioperative outcomes

	Robotic surgeon (*n* = 61)	Robotic surgeon in training (*n* = 63)	*p*
Age			
Female	4 (6.5)	2 (3.2)	0.436
BMI	25.1 (22.6–27.4)	25.6 (22.8–28.9)	0.154
ASA ≥ 2	19 (31.1)	22 (34.9)	0.705
Right hernia	28 (45.9)	34 (53.9)	0.473
Type of hernia			0.709
Direct hernia	23 (37.7)	21 (33.3)	
Indirect hernia	32 (52.4)	36 (57.1)	
Mixed hernia	5 (8.2)	6 (9.5)	
Femoral hernia	1 (1.6)	0 (0)	
Recurrent hernia	7 (11.5)	8 (12.7)	1.000
Hernia classification			0.337
EHS I	25 (41.0)	34 (53.9)	
EHS II	26 (42.6)	20 (31.7)	
EHS III	10 (16.4)	9 (14.3)	
Operative time (min)			
T1	4 (3–5)	5 (3–5)	0.014
T2	30 (22–35)	35 (31–37)	0.001
T3	10 (7–14)	10 (7–15)	0.974
T4	8 (6–12)	8 (6–12)	0.457
90-Days postoperative morbidity	5 (7.9)	4 (6.3)	0.741
Length of hospital stay	1 (1–2)	1 (1–2)	0.618

Preoperative characteristics were similar between the groups. The experienced surgeon showed shorter T1 (4 versus 5 min, *p* = 0.014) and T2 intervals (30 versus 35 min, *p* = 0.001), while the duration of the remaining two steps was similar.

Postoperative outcomes were similar between the groups.

### Learning curve analysis

**Fig. 1:**
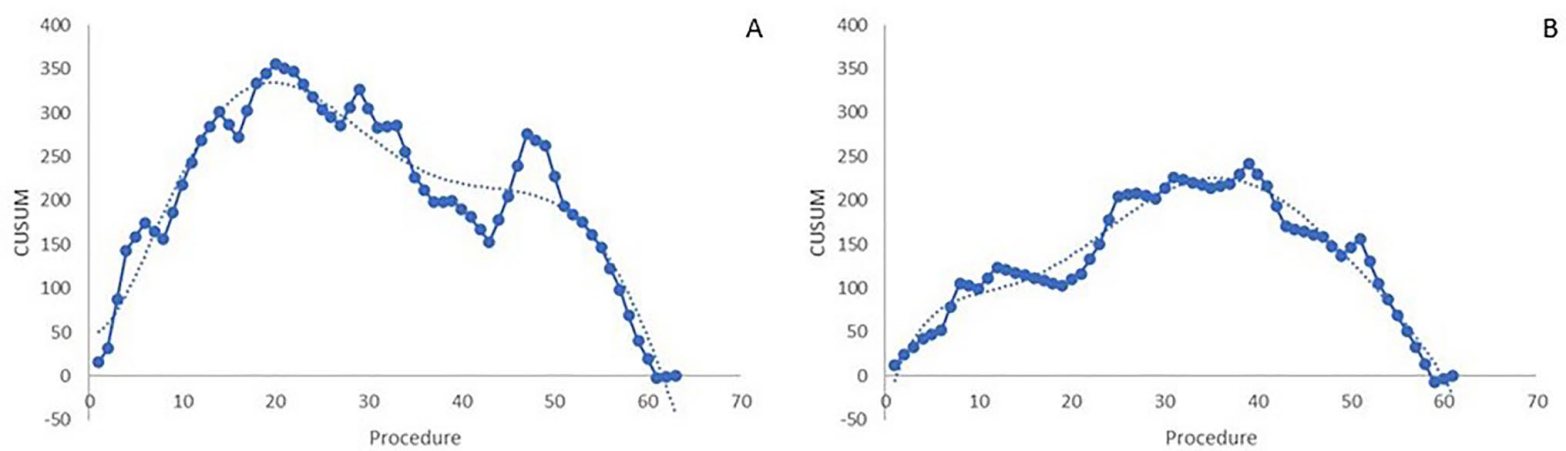
CUSUM charts of skin-to-skin operative time for the surgeon in training with the robotic platform (**a**) and the expert surgeon (**b**). Dotted lines represented trend lines

The CUSUM charts of operative time analyzing the LC of both surgeons are shown in Fig. 1. The surgeon in training with the robotic platform showed a learning phase till the 20th procedure followed by a gradual improvement in performances which are more evident from the 51st procedure (Fig. 1a). Differently, the expert surgeon showed a learning phase till the 35th procedure after which a constant decrease of operative time was recorded till the last procedure included (Fig. 1b).

**Fig. 2:**
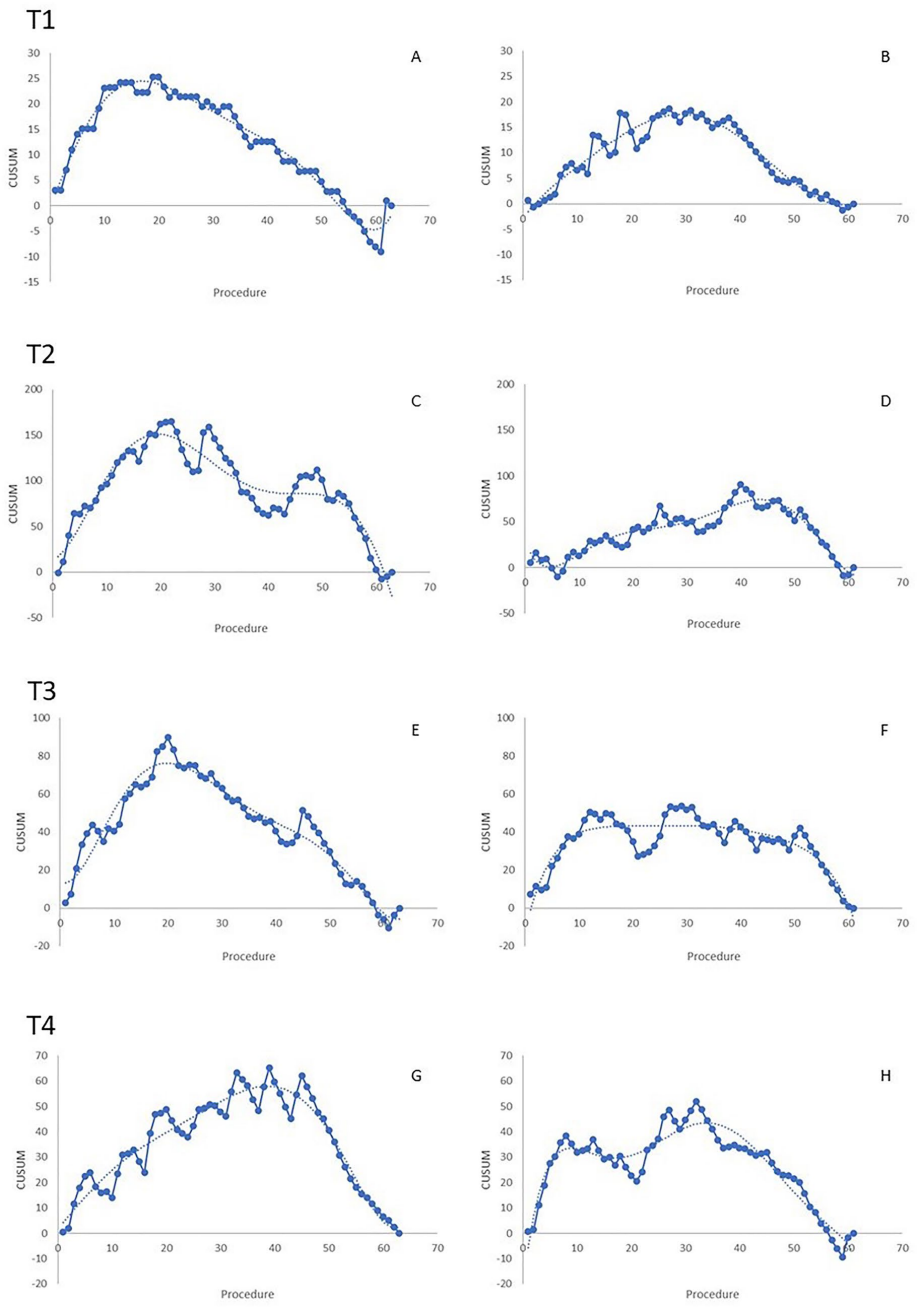
CUSUM charts of T1–T4 operative times for the surgeon in training with the robotic platform (**a**, **b**, **e**, **g**) and the expert surgeon (**b**, **d**, **f**, **h**). Dotted lines represented trend lines

The LC details for T1 are shown in Fig. 2. The graph showed a peak at the 19th and 30th procedure for the surgeon in training (Fig. 2a) and the expert one (Fig. 2b), respectively.

T2 curves had similar characteristics of the charts of global operative times (Fig. 2c, d).

The trend line in the T3 CUSUM graph of the surgeon in training had one peak after 20 cases (Fig. 2e). The expert surgeon’s curve showed a peak after 20 cases followed by a plateau till the 50th procedure (Fig. 2f).

The CUSUM charts of T4 are shown in Fig. 2g, h. The analysis of the CUSUM chart of the surgeon in training showed that 40 procedures were required to acquire proficiency in this phase (Fig. 2g). Differently, the trend line in the CUSUM chart of the expert robotic surgeon had two peaks: the first was around the 8th procedure and the second at the 45th (Fig. 2h).

### Outcomes after learning phase completion

Patients’ characteristics and postoperative outcomes after the learning phase completion are shown in Table 2. No differences were seen in postoperative outcomes between the two surgeons. Learning phase, 90 days of postoperative morbidity, was similar to those after its completion for the trainee (pre-learning 1, 5% versus post-learning 3, 7.0%; *p* = 1.000) and the expert surgeon (pre-learning 5, 16.6% versus post-learning 0, 0%; *p* = 0.066). Details on operative times and differences according to the end of the learning phase of each surgical step per surgeon are presented in Table 3. After the learning phases (after 20 cases for the surgeon in training and after 35 cases for the expert surgeon), the skin-to-skin operative time of the experienced robotic surgeon was significantly shorter than the one of the surgeon in training with the robotic platform (52 min, 45–65 versus 65, 53–75; *p* = 0.0001).

**Table 2:** Patients’ characteristics and postoperative outcomes following the completion of the learning phase

	Robotic surgeon (*n* = 26)	Robotic surgeon in training (*n* = 43)	*p*
Age	68 (48–78)	60 (49–74)	0.216
Female	2 (7.7)	0 (0.0)	0.138
BMI	24.9 (21.6–28.7)	24.7 (21.7–27.0)	0.883
ASA ≥ 2	10 (38.5)	12 (27.9)	0.428
Right hernia	14 (53.8)	21 (48.8)	0.805
Type of hernia			0.66
Direct hernia	11 (42.3)	14 (32.5)	3
Indirect hernia	14 (53.8)	26 (60.5)	
Mixed hernia	1 (3.8)	3 (6.9)	
Femoral hernia	0 (0.0)	0 (0.0)	
Recurrent hernia	4 (15.4)	4 (9.3)	0.464
EHS Hernia classification			0.80
EHS I	11 (42.3)	18 (41.9)	0
EHS II	8 (30.8)	16 (37.2)	
EHS III	7 (26.9)	9 (20.9)	
90-Days postoperative morbidity	0 (0)	3 (7.00)	0.549
Length of hospital stay	1 (1–2)	1 (1–2)	0.978

> 20 cases for the surgeon in training and > 35 cases for the expert surgeon

**Table 3:** Operative times comparison according to the completion of the learning phase of each step

Operative times (min)	Robotic surgeon			Robotic surgeon in training		
	Learning phase	Proficiency phase	*p*	Learning phase	Proficiency phase	*p*
T1	5 (3–5)	3 (3–5)	0.020	5 (5–8)	4 (3–5)	0.0001
T2	31 (25–35)	23 (19–30)	0.015	44 (35–50)	31 (24–37)	< 0.001
T3	12 (10–15)	8 (6–11)	0.002	15 (13–17)	9 (6–12)	< 0.001
T4	13 (11–17)	7 (6–10)	0.001	11 (8–17)	7 (6–8)	0.007
Skin-to-skin	67 (60–75)	52 (45–65)	0.0001	90 (80–105)	65 (53–75)	0.0001

## Discussion

The LC of rTAPP is different according to the surgeon’s experience with the robotic platform.

In our analysis, it was found that the surgeon in training with the robotic platform completed his learning phase around the 20th procedure, which was followed by a gradual improvement while the expert surgeon required 15 cases more to complete his first phase. As expected, the trained surgeon had consistently shorter operative times than the one in training. This was particularly evident in T2, which represented the central and most challenging part of the procedure. The earlier completion of the learning phase of all steps for the surgeon in training should be ascribed to the particularly long time required to perform the very first case. This resulted in a very steep curve at the beginning which quickly reversed the trend in a gradual slope. In contrast to this, the curve of the expert surgeon showed a longer learning phase followed by a sensible decrease of the duration of the different steps. These differences may represent the time to familiarize with the robotic platform which, for its own characteristics, requires only few cases to acquire proficiency. Additionally, it must be noted that the operative time during the proficiency phase of T2 for the surgeon in training was similar to the one of the expert surgeons during the learning phase.

Our outcomes may be difficult to be compared with others reported in literature as the published study on the topic dealt with surgeons who performed at least 150 laparoscopic inguinal hernia repairs before starting with the robotic approach [7, 8]. In particular, Proietti et al. [8] reported data on two surgeons who had no experience with the robotic platform: they found that 43 procedures were required to achieve 90% proficiency in operative time on the logarithmic tendency line. Nevertheless, at a closer look of the LG graph, it seems that the first and only peak of the CUSUM chart appeared in the 9th case which could represent the turning point on the surgeon’s comfort in using the robotic platform. As expected, this occurred earlier than the surgeon in training in this study as he had no experience with rTAPP.

Differently, Kudsi et al. [7] reported the experience with rTAPP of a surgeon, who was an expertise in laparoscopic inguinal hernia repair and completed 40 robotic procedures before starting with rTAPP. As such, in this case, the robot-related LC was partly completed, and this had a noticeable reflection on the reported operative times which were around 30% shorter than the ones reported in our experience both in the early and the late phases. It must be highlighted that this may also be due to the fact that Kudsi et al. reported a series of 371 robotic TAPP performed in a 5-year period during which other robotic procedures were also performed by the same surgeon.

The European hernia society guidelines reported that the plateau in the LC after laparoscopic TAPP could occur after 50–100 cases [13]. A recent systematic review, including six studies, found that the median number of procedures required to overcome the LC for laparoscopic TAPP was 37.5 (range 13–75) [14]. As per the rTAPP, it is more difficult to make such a statement because, as stated above, studies on the topic are limited and heterogeneous in terms of surgeons’ experience both with TAPP and robotic surgery. Our results may be similar to those presented by Ebeling et al. who reported that case loads > 30 in rTAPP in residents with no experience with TAPP or robotic surgery were associated with improved competency and autonomy [15]. This may support the hypothesis that the LC for rTAPP may be slightly shorter than the laparoscopic one. However, it must be highlighted that the proficiency of the residents was defined assessing the association between the GEAR (Global Evaluative Assessment of Robotic Skills [16]) score and the case load without using the CUSUM analysis of the operative time.

In addition, it should be noted that most of the studies about learning curves for inguinal hernia repair with the robotic or laparoscopic approach were characterized by the lack of details about the pre-study skills of the participating surgeons and the level of support they had while in the learning phase. It remains that additional homogeneous data reporting more details about the experience and the pre-existing skills of the participating surgeons are needed in order to confirm our results and to highlight the potential benefit of using the robotic platform in terms of length of the learning phase.

This study has a few limitations. First, the retrospective analyses of the recorded procedures may result in the lack of few details on the technical issues experienced “outside” the robotic platform (e.g. docking, trocar placement, non-surgeon operating room personnel familiarity with the robotic platform). Second, our analysis of LCs was performed on a relatively small number of cases, especially, if compared with those studies on the LC for laparoscopic TAPP. In our opinion, this should be considered when interpreting our results. However, we believe that clinically relevant outcomes related to the learning curves may be mainly linked to the very first part of the learning curves and that late phases may be characterized only by minor adjustments.

Finally, we did not reported details on long-term outcomes, such as recurrent hernia, which could have helped in defining the quality of the repair in the different learning phases.

In conclusion, the learning phase of rTAPP surgery may vary between 20 and 35 cases, depending on the surgeon’s experience in robotic surgery. According to the CUSUM analysis, the expert robotic surgeon could complete the learning phase after 35 procedures. On the other hand, the trainee who had prior basic training in robotic surgery and rTAPP before the study, as well as during the study, could achieve proficiency in rTAPP in approximately 20 cases. The operative times to complete T2 during the proficiency phase of the robotic surgeon in training were comparable to those of the expert surgeon during the learning phase.

## Data Availability

The data that support the findings of this study are available from the corresponding author, [LS], upon reasonable request.
